# The determination of the effect(s) of solute carrier family 22-member 2 (*SLC22A2*) haplotype variants on drug binding via molecular dynamic simulation systems

**DOI:** 10.1038/s41598-022-21291-4

**Published:** 2022-10-08

**Authors:** Zainonesa Abrahams-October, Rabia Johnson, Mongi Benjeddou, Ruben Cloete

**Affiliations:** 1 Precision Medicine Unit, Department of Biotechnology, Faculty of Natural Sciences, University of the Western Cape, Robert Sobukwe Road, Bellville, 7535 South Africa; 2 Biomedical Research and Innovation Platform, South African Medical Research Council, Tygerberg, Cape Town, 7505 South Africa; 3 Division of Medical Physiology, Faculty of Medicine and Health Sciences, Stellenbosch University, Tygerberg, 7505 South Africa; 4 South African Medical Research Council Bioinformatics Unit, South African National Bioinformatics Institute, University of the Western Cape, Private Bag X17, Bellville, Cape Town, 7535 South Africa

**Keywords:** Biological techniques, Biotechnology, Computational biology and bioinformatics, Health care, Medical research

## Abstract

Single nucleotide polymorphisms detected in the solute carrier member family-22 has been shown to result in a variable response in the treatment of type 2 diabetes mellitus with Metformin. This study predicted a three-dimensional protein structure for the *SLC22A2* protein sequence using AlphaFold 2 and modelled five haplotypes within *SLC22A2* protein structure observed in the Xhosa population of South Africa. The protein models were used to determine the effect(s) of haplotype variations on the transport function of Metformin and 10 other drugs by the *SLC22A2* protein. Molecular dynamic simulation studies, molecular docking and interaction analysis of the five *SLC22A2* haplotypes were performed in complex with the ligand 5RE in a POPC lipid bilayer to understand the mechanism of drug binding. Weakest binding free energy was found between 5RE and haplotype 1. Molecular docking studies indicated the top binding ligands as well as Metformin to bind inside the transport channel in all haplotypes increasing the probability of Metformin inhibition during co-administration of drugs. Metformin showed reduced binding affinity and number of interactions compared to the top four binding molecules. Molecular dynamic simulation analysis indicated that haplotypes 1, 3 and 4 were less stable than 2 and 5. The findings suggest haplotypes 4 and 5 having stronger preference for large inhibitor molecule binding in the active site and this could result in haplotypes 4 and 5 demonstrating reduced Metformin clearance via the *SLC22A2* transporter during co-administration of drugs. The current study is the first to investigate the potential effect(s) of haplotype variation on the protein structure of SLC22A2 to assess its ability to transport Metformin in an indigenous South African population.

## Introduction

The incidence of diabetes mellitus (DM) is constantly on the rise across the world. In the African continent, South Africa contributes 7% of DM burden^1^. Type 2 diabetes mellitus (T2DM) accounts for ~ 90% of all DM cases and the preferred first line treatment for the management of T2DM is Metformin. Nonetheless, variability in drug response has resulted in several other classes of drugs being prescribed to treat T2DM, including: sulfonylureas, meglitinides, thiazolidinediones, α-glucosidase inhibitors, dipeptidyl peptidase-4 inhibitors, glucagon-like peptide-1 agonist, sodium glucose cotransporter-2 inhibitors, insulin and its analogues^2–4^.

The variability in response to the treatment of T2DM has been linked to genetic and environmental factors^5,6^. Genetic polymorphisms in candidate genes involved in drug absorption, transportation, distribution, and excretion (ADME) contribute to the observed heterogeneity in the treatment and management of T2DM^2,7^. Genetic variants in the solute carrier family (*SLC*) are amongst the drug related genes that have altered response in the treatment of T2DM and have therefore been selected for further examination^8^.

Members of the *SLC22* family share a common structure of 12 transmembrane domains (TMDs), a large extracellular loop with glycosylation sites between domains 1 and 2, and a intracellular loop with phosphorylation sites between domains 6 and 7^9^. The *SLC22A2* gene encodes for the organic cation transporter 2 (*OCT2*) isoform which acts as a renal uptake transporter^10^. *OCT2* is expressed on the basolateral side of the proximal tubule cells and plays a crucial role in the disposition and renal clearance of cationic drugs and endogenous compounds^11^. *OCT2* is a poly-specific transporter that facilitates the bi-directional transport of structurally unrelated small organic cations down their electrochemical gradients^12,13^. It transports positively charged drugs and since most prescribed drugs belong to the organic cations which are positively charged, the function of it as a transporter has pharmacological consequences.

Many drugs (ligands) have been identified as substrates and/or inhibitors of *SLC22A2* including the oral anti-diabetic drug Metformin and the chemotherapeutic agent Cisplatin^14–16^. Metformin (C_4_H_11_N_5_) has a molecular weight of 129.16 g/mol, whilst Cisplatin (Cl_2_H_6_N_2_Pt) which is larger, has a molecular weight of 300 g/mol^17^. Larger ligands are more likely to interact with more residues at the same binding pocket of a protein than smaller ligands inferring that the protein would preferentially bind larger ligands. However, smaller ligands can have more options in binding pockets on a protein structure. Furthermore, the binding of ligands to proteins are further affected by amino acid (aa) changes^18^. Amino acid (aa) residue(s) substitutions/mutations can affect protein conformation and the function of the protein regardless of their location^18^. Since single nucleotide polymorphisms (SNPs) are most likely to occur in combinations, the incorporation of haplotypes in pharmacogenetic studies is becoming more relevant in the practice of genetic medicine at both an individual and population level^19^.

A computational approach, using molecular docking, was previously used to determine the effect(s) of the common non-synonymous SNP (nsSNP) rs316019 (A270S) on the interaction of Metformin and other drugs within the *SLC22A2* protein^20^. The study determined that the WT protein was more efficient at ligand binding than the variant since it had a more open and wider space in its binding pocket^20^. In addition, the study also explored the two haplotypes, i.e. WT and the A270S variant. The current study aims to explore these haplotypes including additional haplotypes which have been identified in a sub-Saharan African population, i.e. the Xhosa population of South Africa. This study will determine if the haplotypes identified in the Xhosa population of a South African cohort could alter the transport function of *SLC22A2*. This will be achieved by building structural models for each of the five haplotype sequences that have been observed in the Xhosa population. Molecular dynamic simulation studies were used to understand the difference in the binding of inhibitor 5RE to the five haplotype structures in a POPC lipid bilayer. Furthermore, molecular docking and interaction analysis of *SLC22A2* substrates &/or inhibitors (i.e. Cimetidine, Creatinine, Dolutegravir, Isavuconazole, Metformin, Ranitidine, Ranolazine, Trimethoprim and Vandetanib) will provide novel insights into the mechanism of binding of these substrates and inhibitors to *SLC22A2*.

## Materials and methods

### SNP frequency and haplotype structures of *SLC22A2*

Previously, Jacobs et al. (2015) characterised the frequency of various haplotypes in the *SLC22A2* gene that exists within the South African Xhosa population^21^. Three nsSNP, i.e. rs316019 results in aa substitution (Ala270Ser), rs8177516 in aa substitution (Arg400Cys) and rs8177517 in aa substitution (Lys432Gln), were detected. These three nsSNPs characterises the five identified haplotypes which were found in the study cohort.

### Change in phenotype predictions using SIFT, PolyPhen 2 and I-mutant

The phenotypic effects of three aa substitutions, (i.e. Ala270Ser, Arg400Cys and Lys432Gln), on the structure and function of *SLC22A2* protein sequence was investigated using a variety of algorithms that included: the Sorting Intolerant from Tolerant (SIFT)^22–24^, the Polymorphism Phenotyping 2 (PolyPhen-2)^25–27^ and the I-mutant algorithms^28^. The SIFT program uses sequence homology to predict the potential effect(s) an aa substitution could have on protein function^22^. It calculates a tolerance index score ranging from 0 (deleterious) to 1 (neutral) based on multiple sequence alignments. Variants with scores ranging from 0.00 to 0.049 are considered deleterious, with scores closer to 0.00 being more confidently predicted as deleterious. Variant scores ranging between 0.05 and 1.00 are predicted as neutral or tolerated with scores closer to 1.00 more confidently predicted as neutral. PolyPhen-2 predicts the possible impact of an aa substitution on protein structure and function by physical and evolutionary comparisons^27^. It categorizes aa substitutions from 0 (neutral) to 1 (deleterious). In addition, it also provides the functional significance of each aa substitution as: benign (0.00–0.14), possibly damaging (0.15–0.84) and probably damaging (0.85–1). The I-mutant algorithm, predicts the effect(s) of single point mutations on protein folding using a support vector machine-based approach that includes either sequence or structural information^28^. The predicted I-mutant score is a reliability index ranging from 0 (least likely to result in the altered protein stability as predicted) to 8 (most likely to result in the altered protein stability as predicted)^28,29^.

### Amino acid sequence retrieval, molecular modelling of *SLC22A2* (O15244) and structure quality assessment

The *SLC22A2* primary aa sequence was retrieved and downloaded from the UniProtKB database with the accession number O15244. The protein structure of *SLC22A2* has not been experimentally resolved. Therefore homology modelling methods were employed to predict the three-dimensional (3D) structure of *SLC22A2* using the online 3D structure prediction webserver AlphaFold 2 Protein Structure Database^30,31^. AlphaFold 2 is an artificial intelligence system developed to predict the 3D structure of a protein from its aa sequence^30^. It constructs a multiple sequence alignment by comparing the input amino acid sequence to several protein sequence databases. AlphaFold 2 identifies similar sequences to the input aa sequence and extracts the information to produce an accurate 3D structure model^30^.

The generated protein model was further assessed using internal scores to validate the quality of the predicted model. Since the *SLC22A2* protein is a transmembrane protein, the local model quality estimation method for membrane proteins (QMEANBrane) score was determined^32^. The QMEANBrane score is a version of QMEAN developed to assess the local quality of alpha-helical transmembrane protein models^32^. It takes into the consideration the three different segments in a transmembrane protein (membrane, interface and soluble) model. Values are normally between 1 and 0, with higher values indicating more reliable models^32^. To determine if the correct fold/conformation was assigned to the protein sequence a structural alignment was performed to calculate a root mean square deviation (RMSD). The lower the RMSD value, the closer the structures are in terms of backbone deviation. External quality analysis was done using the Structure Assessment and Verification Server (SAVES) webserver and included tools such as ERRAT and Procheck. ERRAT statistically interrogates the non-bonded atomic interactions of the given protein model against the interactions of well-refined structures^33^. Higher overall quality factors of 80% and more suggest very few errors within the protein model, suggesting it is highly reliable. Procheck generates a Ramachandran plot and determines if the phi and psi dihedral angle distribution of protein residues satisfy conformations found in crystal structures of high resolution^34,35^. Usually, more than 90% of residues in a protein structure needs to be located in the most favourable regions of the plot to be considered an accurate protein structure.

### Structure preparation for molecular dynamic simulation studies

Five simulation systems (i.e. haplotypes 1 to 5) were prepared in total for *SLC22A2* in complex with inhibitor (2~{*S*}-3-(4-fluorophenyl-2-[2-(3-hydroxyphenyl)ethanoylamino]-~{*N*}-[(1~{*S*}-1-phenylethyl] propenamide (PDBID: 5RE) using Pymol by aligning the template structure and *SLC22A2* predicted structure and extracting the compound 5RE^36^. The variant haplotype structures were prepared using the PyMol mutagenesis wizard where: haplotype 1 (WT) had no aa change, haplotype 2 (A270S), haplotype 3 (A270S and R400C), haplotype 4 (A270S and K432Q) and haplotype 5 (R400C). The five haplotype complex systems were embedded into a heterogeneous lipid bilayer consisting of 120 1-palmitoyl-2-oleoylphosphatidylcholine (POPC) lipids. The lipid bilayer consisted of 60 lipids in the upper leaflet and 60 in the lower leaflet with a water thickness of 22.5 Å from the top leaflet and the bottom leaflet. The POPC lipid bilayer is one of the most abundant lipids found in human transmembrane proteins. The CHARMM-GUI membrane builder was used to orient the protein relative to the POPC lipid bilayer membrane^37^. The proteins of each system were aligned along the first principal axis the Z-axis of the membrane with the protein in the center (Z = 0). All five simulations were performed using the GROMACS-2019 package^38^ along with the CHARMM36M all-atom force field^39^. The accurate topologies for the ligand was generated using ParamChem/CHARMM general force field^40^. All the systems were solvated with TIP3 water molecules in a cubic box of at least 10 Å of water between the protein and edges of the box at a concentration of 0.15M. To neutralize the positive and negative charges of the systems for haplotype one and two, 63 potassium (K) ions and 70 chloride (Cl) ions were added to neutralize the charge of the system. While, haplotype three, four and five had 62 K ions and 68 Cl ions, respectively.

Each system underwent 50,000 steps of steepest descents energy minimization to remove close Van der Waals force contacts. Subsequently, all systems were subjected to a two-step equilibration phase namely; NVT (constant number of particles, Volume and Temperature) for 500 ps to stabilize the temperature of the system and a short position restraint NPT (constant number of particles, Pressure and Temperature) for 500 ps to stabilize the pressure of the system by relaxing the system and keeping the protein restrained. For the NVT simulation the system was gradually heated by switching on the water bath and the V-rescale temperature-coupling method was used, with constant coupling of 0.1 ps at 300 K under a random sampling seed. While for NPT the Parrinello-Rahman pressure coupling^41^ was turned on with constant semi-isotropic pressure coupling for uniform scaling of x, y box vectors of 0.1 ps at 300 K under conditions of position restraints (all-bonds). For both NVT and NPT, electrostatic forces were calculated using the Particle Mesh Ewald method^42^ and all systems were subjected to a full 200 ns simulation.

### Trajectory analysis, non-bonded interaction energy and lipid bilayer analysis

The analysis of the trajectory files was done using GROMACS utilities. The root-mean square deviation (RMSD) was calculated using gmx rmsd for the protein back bone atoms and the root mean square fluctuation (RMSF) analysis for the protein residues was calculated using gmx rms. The average number of hydrogen bonds was calculated using the gmx hbond tool, while the distance between the drug and the protein was calculated using gmx pairdist. The non-bonded interaction energy was calculated between the protein and the drug using (gmx energy) and the free energy of binding was calculated between the protein and the drug 5RE using the Molecular Mechanics Poisson-Boltzmann Surface Area (MMPBSA) protocol implemented in g_mmpbsa package over the last 300 frames of the simulation trajectory^43^. We characterized the simulated POPC lipid bilayer in complex with the protein by calculating several parameters. This lipid bilayer analysis comprised of different calculations with the corresponding GROMACS utility indicated in parentheses: the area per lipid (gmx energy) to analyze the X and Y box dimensions, the bilayer thickness (gmx density) for the phosphate atom headgroups, the lateral diffusion coefficients (gmx mean square displacement (msd)) for the lipid head groups, the deuterium order parameters for all carbons in the acyl chains was calculated using g_lomepro^44^. The average number of hydrogen bonds were also calculated between the protein and POPC lipids (gmx hbond), and the non-bonded interaction energy (gmx energy) between the protein and the POPC lipid. The final snapshot of the simulation systems was extracted at 200 ns and were visually illustrated using PyMol.

### Molecular docking of inhibitors and interaction analysis

The equilibrated structural conformations were extracted at 40 ns simulation time period for each of the simulation trajectories using gmx trjconv tool. Eleven compounds, including 5RE were docked to the conformational snapshot structures. The eleven drug structures (ligands) were retrieved from Pubchem^17^. They were selected because they were identified as known cationic compounds to be transported by *SLC22A2*^11^. Metformin (a *SLC22A2* substrate), Glibenclamide and Gliclazide (substrates of *CYP2C9*) are commonly prescribed for the treatment and management of T2DM^45,46^. The remaining ligands, (i.e. Dolutegravir, Isovuconazole, Vandetanib, Ranolazine, Trimethoprime, Cimetidine and Creatinine) are drugs prescribed in the treatment of a range of diseases and illnesses, and are known to be substrates &/or inhibitors of *SLC22A2*. Co-administration of drugs can influence the efficacy and action of the anti-diabetic drug Metformin.

Molecular docking of the drug substrate 5RE and the selected 11 ligands to the five haplotype variant structures of the *SLC22A2* protein was performed using Autodock Vina^47^. All haplotype receptors were prepared using AutoDock tools, which add polar hydrogens, Gasteiger charges and saves the output file in pdbqt format. The twelve ligands were prepared with automated bash and python scripts namely, split_multi_mol2_file.py and prepare_ligand4.py^48^. This was done to correct for errors such as missing atoms, added H_2_O, more than one molecule chain break and alternate locations. After receptor and ligand preparations, the centre of mass was calculated for the ligand 5RE in complex with each of the receptor structures to determine the X, Y and Z coordinates of the docking grid space using a python script center_of_mass.py. Various parameters for the docking process were stored in a configuration file. The configuration file contained all the input parameters for all docking simulations, such as: centre of mass coordinates, grid space and exhaustiveness of the search algorithm (grid box dimensions are provided in Table 1). Interaction analysis was performed using PoseView web^31^. The docked complexes were ranked according to their energy scores using the python script developed by the Scripps Research Institute vina_screen_get_top.py. All the docked poses were visually inspected in PyMol and were further analysed using PoseView^31,49^. PoseView determines four types of interactions namely; (i) hydrogen bonds, (ii) hydrophobic, (iii) metal interactions and (iv) π-stacking interactions.

**Table 1:** Docking grid box dimensions used for each haplotype.

	Haplotype 1	Haplotype 2	Haplotype 3	Haplotype 4	Haplotype 5
Center-X	33.4	26.8	30.3	34.5	36.2
Center-Y	34.4	25.4	25.6	40.6	37.5
Center-Z	73.7	78.1	80.7	78.9	76.1
Size-X	24	28	28	28	28
Size-Y	24	26	26	26	26
Size-Z	26	24	24	24	24
Exhaustiveness	30	30	30	30	30

## Results

### Haplotype characterisation

**Figure 1:**
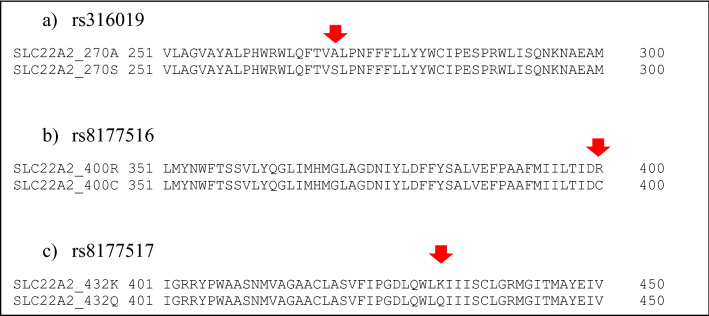
Pairwise protein sequence alignments of *SLC22A2* variants: (**a**) rs316019 (A270S), (**b**) rs8177516 (R400C) and (**c**) rs8177517 (K432Q) (variant position indicated by red arrow).

*SLC22A2* gene was characterised to contain five haplotypes in the South African Xhosa population. Haplotype structure and frequency within the study population is shown in Table 2. The five identified haplotypes are defined by three nsSNPs, i.e. rs316019 (A270S); rs8177516 (R400C) and rs8177517 (K432Q). Pairwise sequence alignments of *SLC22A2* protein and the identified variants are shown in Fig. 1.

**Table 2:** Haplotype structure and frequency of *SLC22A2* variants in a South African Xhosa population.

Haplotype name	SNP combinations	SNP consequence	Frequency (%)
Haplotype 1: WT	N/A	Ala: Arg: Lys	90.0
Haplotype 2	rs316019	Ser: Arg: Lys	6.0
Haplotype 3	rs316019:rs8177516	Ser: Cys: Lys	3.2
Haplotype 4	rs316019:rs8177517	Ser: Arg: Gln	0.7
Haplotype 5	rs8177516	Ala: Cys: Lys	0.1

*WT* wild type, *N/A* not applicable.

Amino acid abbreviations: *Ala* alanine, *Arg* arginine, *Lys* lysine, *Ser* serine, *Cys* cysteine, *Gln* glutamine.

The functional effects of the identified aa substitutions on protein structure and function were assessed using SIFT, PolyPhen-2 and I-mutant algorithms (Table 3). Both SIFT and PolyPhen-2 predicted that the function of *SLC22A2* would be affected by the presence of the R400C variant, whilst the protein function would most likely not be affected by the presence of the K432Q variant. However, these algorithms showed contradictory results for the remaining A270S variant. The I-mutant algorithm which measures the degree of protein destabilization predicted that all of the identified aa substitutions would decrease protein stability.

**Table 3:** Phenotypic effects predicted for aa substitutions identified in a South African Xhosa population on the structure and function of *SLC22A2* using SIFT, Polyphen-2 and I-mutant algorithms.

SNP ID	Amino acid change	SIFT result	SIFT score	PolyPhen-2 result	PolyPhen-2 score	Protein stability	I-mutant score
rs316019	A270S	Deleterious	0.033	Possibly damaging	0.252	Decreased	8
rs8177516	R400C	Deleterious	0.020	Probably damaging	0.987	Decreased	8
rs8177517	K432Q	Tolerated	0.344	Benign	0.122	Decreased	2

Aa one-letter code: *A* alanine, *S* serine, *R* arginine, *C* cysteine, *K* lysine, *Q* glutamine.

SIFT score: 0 = deleterious, 1 = neutral/tolerated; PolyPhen-2 score: 0–0.14 = benign, 0.15–0.84 = possibly damaging, 0.85–1 = probably damaging.

### Molecular modelling and structure validation

**Figure 2:**
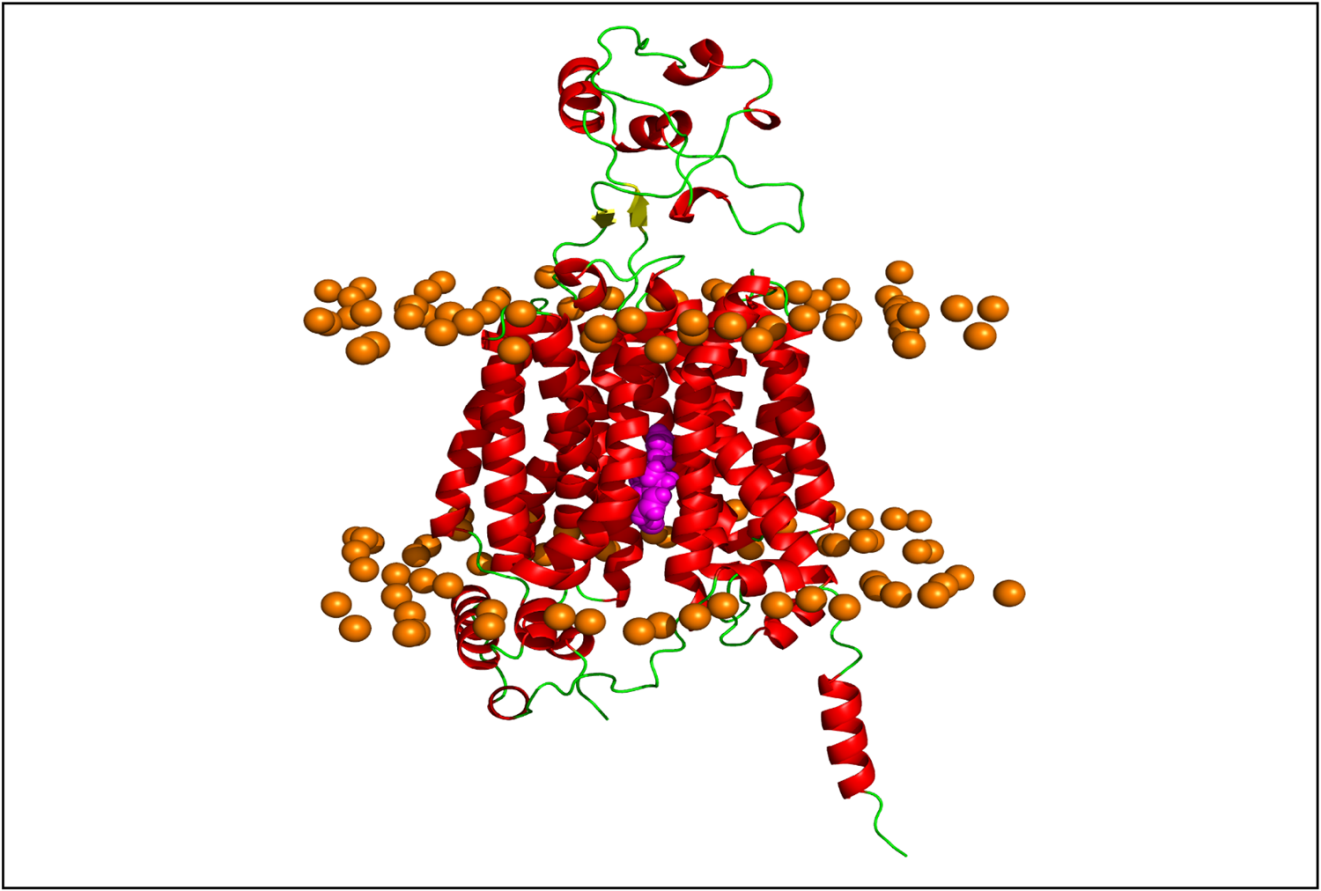
3D structure of *SLC22A2* protein embedded in the POPC lipid bilayer membrane: the *SLC22A2* protein structure (coloured by secondary structure) in complex with the 5RE inhibitor (shown as spheres in magenta) phosphate lipid head groups shown as orange spheres.

**Figure 3:**
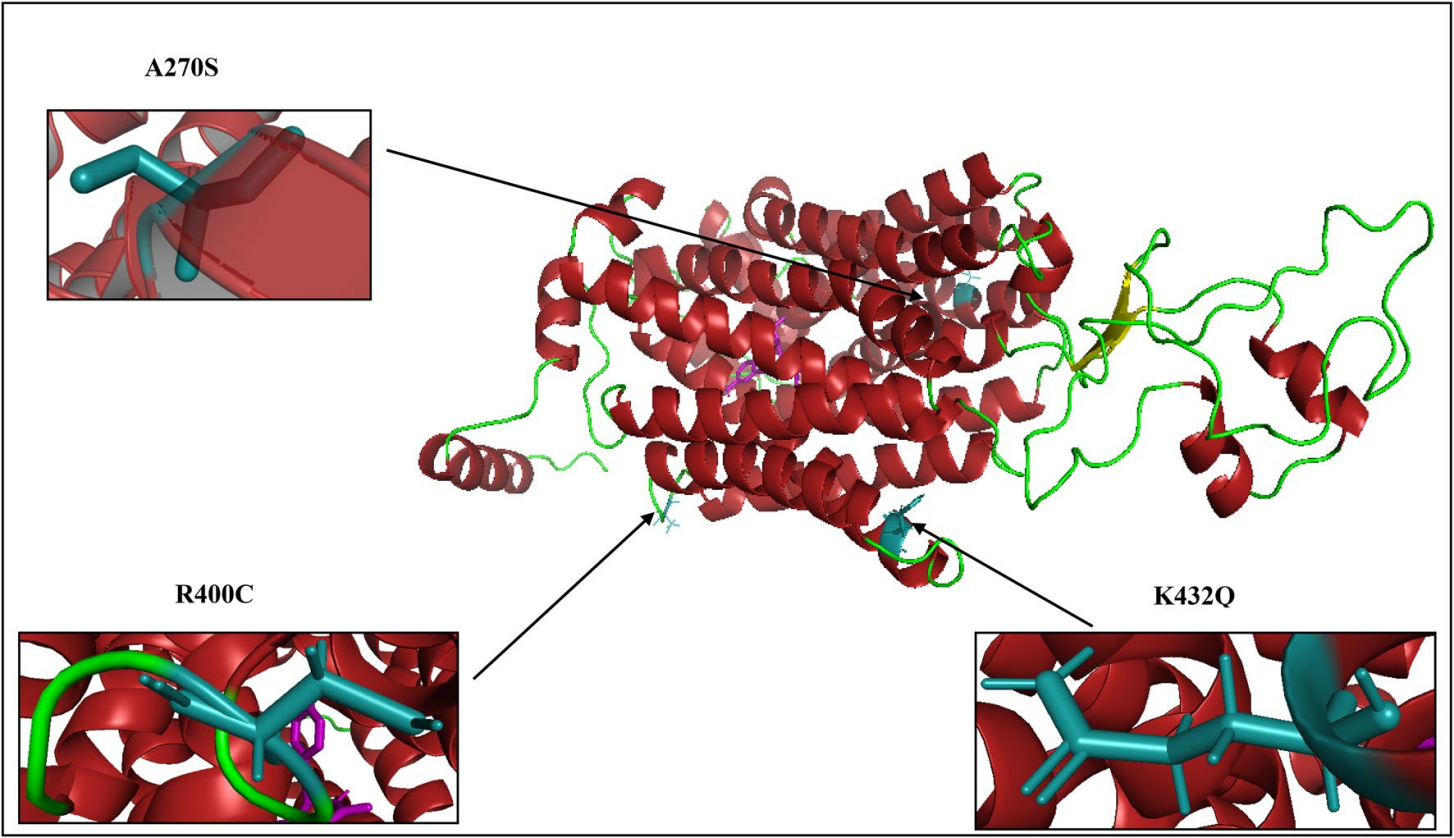
3D structure of *SLC22A2* in complex with 5RE generated by AlphaFold 2: the three mutated residues identified in the South African Xhosa population are shown as sticks (coloured deep-teal) and the 5RE inhibitor in magenta. Solid black arrows point to the locations of the mutations identified to characterise the haplotypes existing in the study population.

A 3D structure was generated by AlphaFold 2 for the *SLC22A2* protein (Fig S1). Figure 2 shows the outward facing conformation of the 3D structure of *SLC22A2* and Fig. 3 shows the locations of the three variants relative to the drug binding site identified in the South African Xhosa population. The overall topology (consisting of alpha helices and beta sheets) of *SLC22A2* is representative of a transmembrane protein involved in drug transport. The QMEANBrane predicted the generated template to be within the expected range (0–1) for a transmembrane structure. The Ramachandran plot indicated that 94.4% of residues had favourable dihedral angle conformations and the ERRAT overall quality factor score was 97.55% suggesting only ~ 2% error rate within the protein model.

### Molecular dynamic simulations and MMPBSA analysis

**Figure 4:**
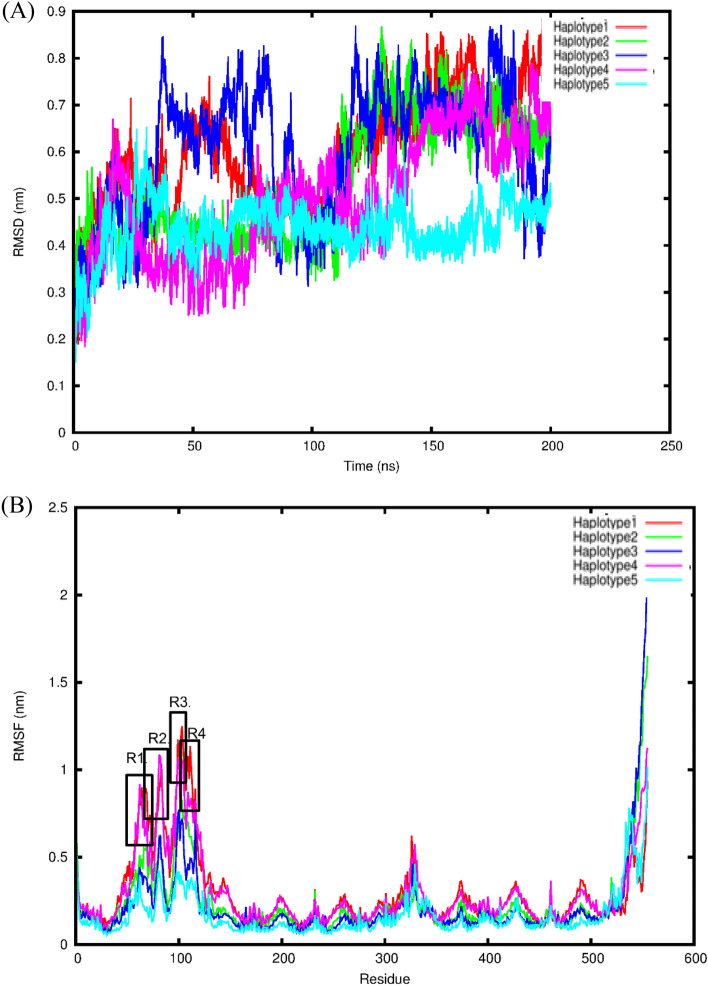
Statistical analysis of haplotypes over 200 ns simulation. (**A**) The backbone RMSD deviation of *SLC22A2*-5RE in the five observed haplotypes [ordinate is RMSD (nm) and the abscissa is time (ns)]. (**B**) RMSF deviation for protein residues for the five haplotype systems over the last 100 ns. Regions of high flexibility are boxed in red and labelled as R1, 2, 3 and 4 on the figure.

All five *SLC22A2*-5RE haplotype systems reached equilibrium after 30 ns based on the backbone RMSD values (Fig. 4A). The mean and standard deviation values for the change in protein RMSD backbone atoms for haplotype 1 (WT-5RE), haplotype 2, 3, 4 and 5 were 0.60 ± 0.12 nm, 0.54 ± 0.13 nm, 0.60 ± 0.14 nm, 0.50 ± 0.13 nm and 0.44 ± 0.06 nm, respectively (Fig. 4A). The RMSD values were the highest for the haplotype systems 1 and 3 compared to haplotype systems 2, 4 and 5. Furthermore, the RMSF fluctuation values were the lowest for haplotypes 5, and 3 each having 0.17 ± 0.12 nm, 0.22 ± 0.23 nm, while haplotypes 2 had an RMSF value of 0.24 ± 0.21 nm compared to haplotypes 1 and 4 each having the largest RMSF value of 0.29 ± 0.22 nm (Fig. 4B). The protein residues of haplotypes 1 and 4 showed four regions (residues R1:62-65, R2:80-85, R3:99-106 and R4:108-113), of high flexibility, while none of the other haplotypes showed any regions of high flexibility (Fig 4B). Interestingly, none of the mutated residues and the most important binding site residues showed high flexibility values, suggesting the active site is stable and not undergoing large conformational changes (Fig.4B).

The distance values between the protein residues and 5RE did not show any significant differences between the five systems. The average number of hydrogen bonds formed between *SLC22A2*-5RE was the highest for haplotype 2 with 2.29 contacts being formed compared to haplotypes 1, 4, 5 and 3 each having 1.53, 1.07, 0.19 and 0.15 bonds, respectively. The non-bonded interaction energy calculated between the protein and the drug was the highest for haplotype 1 (−261.67 ± 42.14 kJ/Mol) followed by haplotype 2, 4 and 3 each having −237.85 ± 30.62 kJ/Mol, −217.18 ± 35.93 kJ/Mol and −215.40 ± 39.80 kJ/Mol while haplotype 5 had the lowest non-bonded interaction energy with a score of −166.35 ± 36.17 kJ/Mol. However, contrasting results were observed for the binding free energy calculations for the different complexes. Here, the total free energy of binding calculated for haplotype 5 was the highest with −106.05 ± 14.32 kJ/Mol, followed by haplotype 4 having a total binding free energy score of −96.79 ± 14.86 kJ/Mol (Table 4). Haplotypes 3 and 2 demonstrated total binding free energies of −93.62 ± 1.74 kJ/Mol and −92.74 ± 14.96 kJ/Mol respectively, while haplotype 1 had the weakest total binding free energy with 5RE of −85.69 ± 14.05 kJ/Mol (Table 4). In summary, the inhibitor 5RE bound the strongest to haplotypes 5 and 2 compared to haplotype 1 (WT) (Table 4).

**Table 4:** MMPBSA energy parameter contributions to the total binding free energy.

Haplotype	van der Waals energy (kJ/mol)	Electrostatic energy (kJ/mol)	Polar solvation energy (kJ/mol)	SASA energy (kJ/mol)	Total ΔG bind protein-5RE (kJ/mol)
1	−195.38 ± 8.61	−48.62 ± 7.75	180.89 ± 14.90	−22.58 ± 0.91	−85.69 ± 14.05
2	−196.83 ± 8.91	−56.34 ± 14.64	183.05 ± 15.90	−22.62 ± 0.93	−92.74 ± 14.96
3	−181.58 ± 11.93	−35.32 ± 8.43	145.50 ± 11.93	−22.23 ± 1.09	−93.62 ± 10.74
4	−209.34 ± 9.54	−47.15 ± 7.37	182.78 ± 13.86	−23.09 ± 0.86	−96.79 ± 14.86
5	−183.79 ± 9.81	−32.41 ± 8.25	132.13 ± 17.43	−21.98 ± 0.94	−106.05 ± 14.32

**Figure 5:**
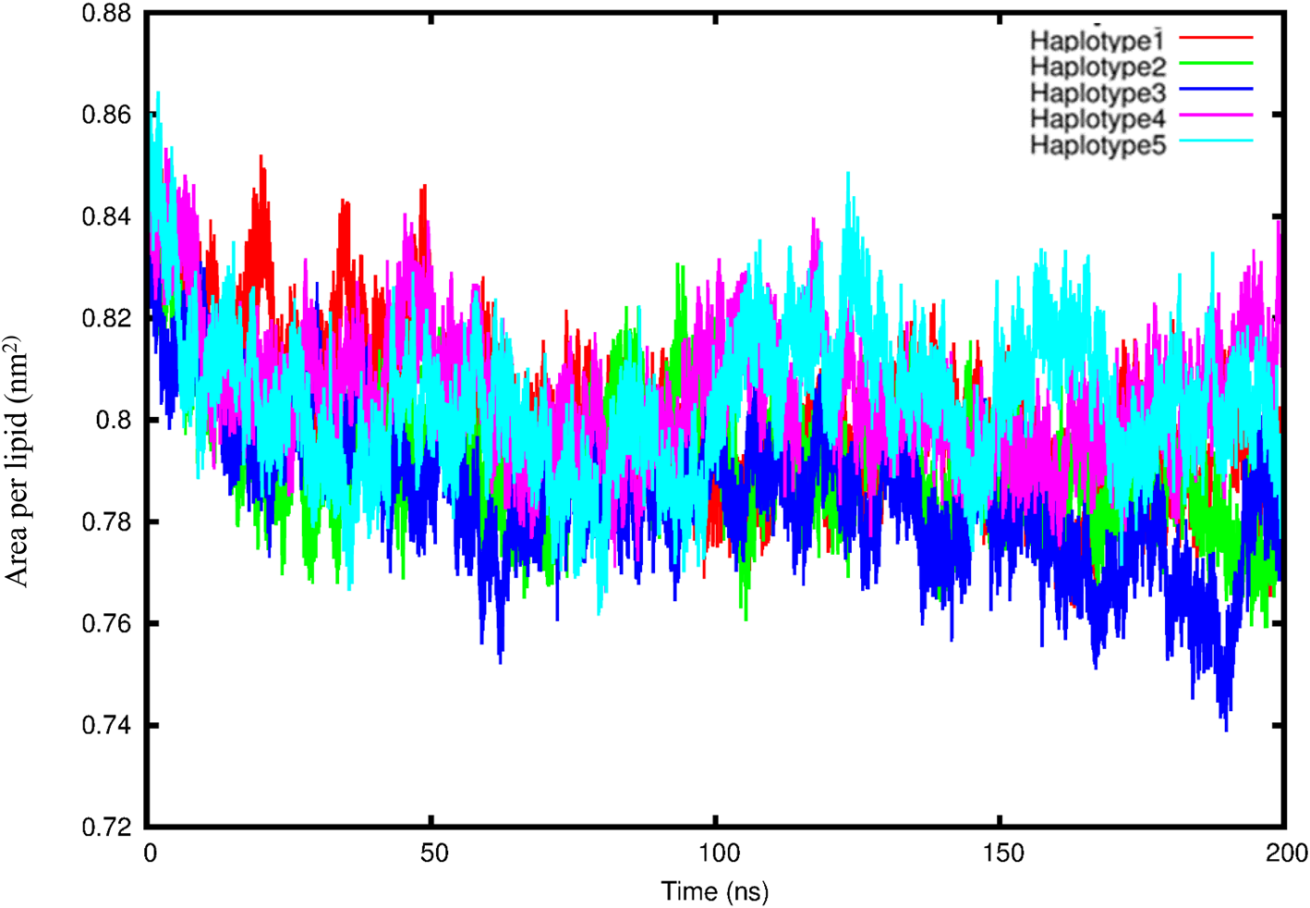
Area per lipid fluctuation for the five haplotype systems over 200 ns.

The average area per lipid reached convergence and fluctuated around a stable mean and standard deviation values for each system ranging between 0.79 ± 0.01 and 0.81 ± 0.01 nm (Fig. 5 and Table 5). The means and SDs for each of the various lipid bilayer properties are summarized in Table 5. The bilayer thickness values was found to shift towards smaller values for haplotype 4 (8.42 ± 4.91 nm) followed by haplotype 5 (8.89 ± 5.18 nm), then haplotype 3 had the largest values (9.02 ± 5.26 nm) and haplotype 2 (8.90 ± 5.18 nm), compared to haplotype 1 having (8.89 ± 5.18 nm). The lateral diffusion coefficient was the lowest for the haplotype 3 (0.86 ± 0.28 cm^2^/s) system, followed by haplotype 5 (1.01 ± 0.22 cm^2^/s), then haplotype 1 (1.16 ± 0.20 cm^2^/s) and haplotype 4 (1.31 ± 0.20 cm^2^/s), while haplotype 2 had the highest, reaching 1.33 ± 0.14 cm^2^/s.

**Table 5:** The lipid bilayer parameters of the 1-palmitoyl-2-oleoylphosphatidylcholine (POPC) simulated lipid bilayer and hydrogen bonds formed between the protein and the POPC lipids.

Haplotype	Area per lipid (nm^2^)	Bilayer thickness (nm)	Lateral diffusion coefficient (cm^2^/s)	Hydrogen bonds
1/WT	0.80 ± 0.01	8.89 ± 5.18	1.16 ± 0.20	22.87
2	0.79 ± 0.01	8.90 ± 5.18	1.33 ± 0.14	27.90
3	0.79 ± 0.01	9.02 ± 5.26	0.86 ± 0.28	27.51
4	0.81 ± 0.01	8.42 ± 4.91	1.31 ± 0.20	27.81
5	0.80 ± 0.01	8.89 ± 5.18	1.01 ± 0.22	14.71

The deuterium order parameters for the POPC carbon acyl chains sn-1 and sn-2 indicated similar results (Figs. S2–S6). For all the systems of chain 1, atoms 4 and 5 had the lowest deuterium order parameter values while atoms 2, 3, 6–8 had higher values, suggesting that atoms 2, 3, 6–8 are not undergoing any large structural changes. For chain 2, the atoms for haplotypes 2, 3, 4 and 5 showed similar results with terminal atoms (6 and 7) had lower deuterium order parameter values, while the central atoms 2–5 had higher values, suggesting that the central atoms are maintaining their structure, while haplotype 1 displayed low deuterium order parameter values for atoms 3, 4 and 6 and higher values for atoms 2, 5, 7 and 8. Calculating the average number of hydrogen bonds formed between the protein and POPC lipids indicated that haplotype 2 formed 27.90, haplotype 4 formed 27.81 and haplotype 3 formed 27.51. Haplotype 5 formed the least amount of hydrogen bonds compared to haplotype 1 each having 14.71 and 22.87, respectively. Interestingly, the non-bonded interaction energy results were similar to the hydrogen bond analysis with haplotype 5 showing the weakest total energy of −4727.42 ± 380.49 KJ/Mol and haplotypes 4 and 2 the largest values of −5031.12 ± 354.91 KJ/Mol and −4998.96 ± 391.41 KJ/Mol compared to haplotype 1 having −4982.29 ± 363.99 KJ/Mol. The final 200 ns snapshot structures for the five haplotype conformations were overlaid and showed similar outward facing conformations for the inner membrane structures with the largest difference being in the outer head region of the protein (Fig. S7).

### Molecular docking studies and interaction analysis

The molecular docking of the 11 drugs to the equilibrated structures at 100 ns for the five structures were performed to determine any difference in drug binding affinity and number of interactions between the different haplotypes. Some drugs are known anti-diabetic compounds such as Metformin, Glibenclamide and Gliclazide. The remaining ligands, (i.e. Cimetidine, Creatinine, Dolutegravir, Isavuconazole, Ranitidine, Ranolazine, Trimethoprim and Vandetanib), can be classified as substrates &/or inhibitors of *SLC22A2*. In haplotypes 1 and 2, four drugs (Isavuconazole, Dolutegravir, Glibenclamide and Gliclazide), whilst in haplotypes 3 and 4 (Glibenclamide, Dolutegravir, Isavuconazole and Vandetanib), in haplotype 5 (Glibenclamide, Dolutegravir, Isavuconazole and Ranolazine) and 5RE consistently ranked among the top binders based on higher binding affinity and number of interactions compared to Metformin that ranked second lowest (Tables 6, 7, 8, 9 and 10). Creatinine demonstrated the lowest docking scores for all five haplotypes.

**Figure 6:**
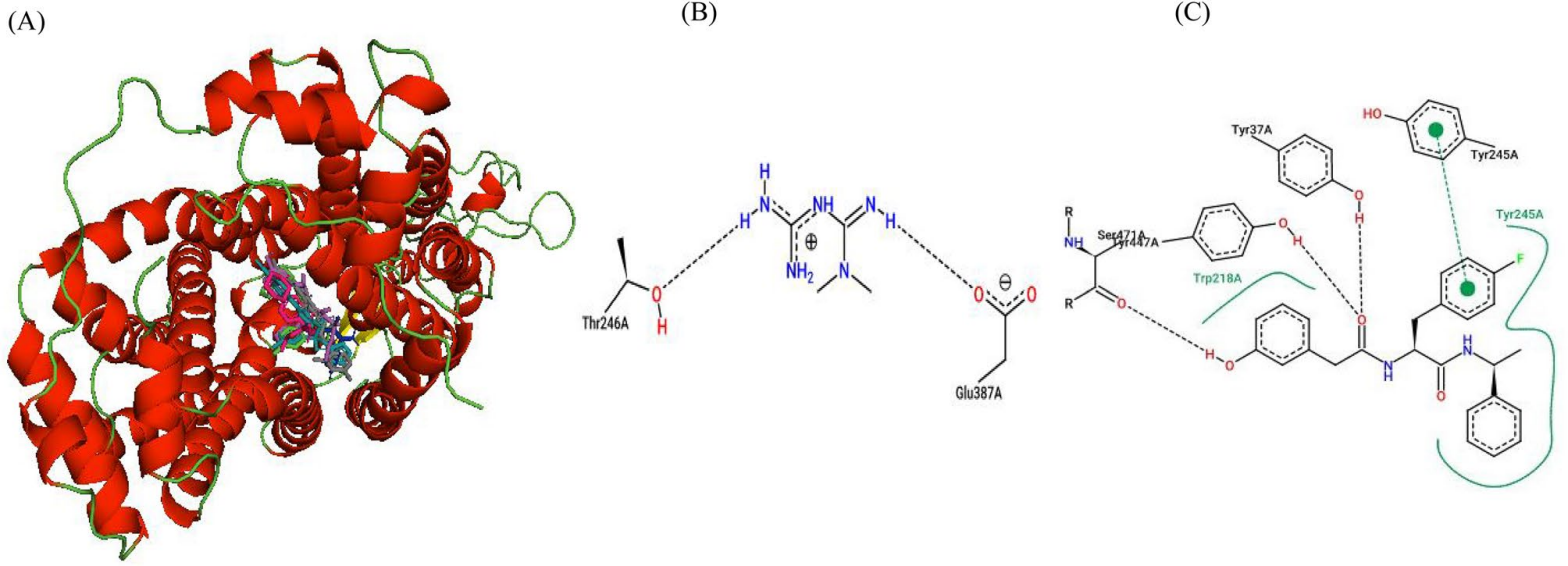
Ligand overlay for haplotype 1 *SLC22A2* protein. (**A**) Image was generated using Pymol with the first line anti-diabetic drug, Metformin, shown in blue. The four top binding ligands Isavuconazole, Dolutegravir, Glibenclamide, and Gliclazide are shown in deep teal, grey, hot-pink and green respectively. The 5RE inhibitor is indicated in magenta. (**B,C**) 2D PoseView illustration, showing interacting residues for Metformin and the 5RE inhibitor respectively—dotted black lines denote hydrogen bonds, solid green lines denote hydrophobic interactions and dotted green lines denote π stacking &/or aromatic interactions.

**Figure 7:**
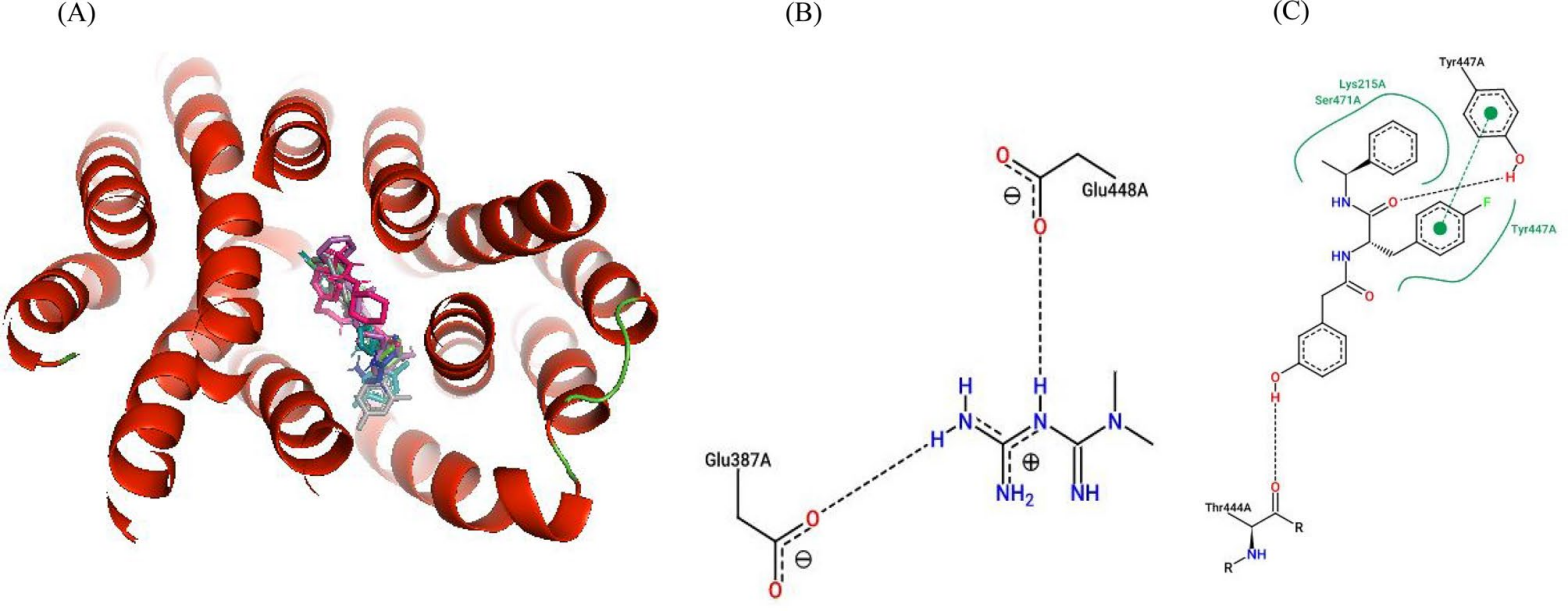
Ligand overlay for haplotype 2 *SLC22A2* protein. (**A**) Image was generated using Pymol with the first line anti-diabetic drug, Metformin, shown in blue. The four top binding ligands Isavuconazole, Dolutegravir, Glibenclamide, and Gliclazide are shown in deep teal, grey, hot-pink and green respectively. The 5RE inhibitor is indicated in magenta. (**B,C**) 2D PoseView illustration, showing interacting residues for Metformin and the 5RE inhibitor respectively—dotted black lines denote hydrogen bonds, solid green lines denote hydrophobic interactions and dotted green lines denote π stacking &/or aromatic interactions.

**Figure 8:**
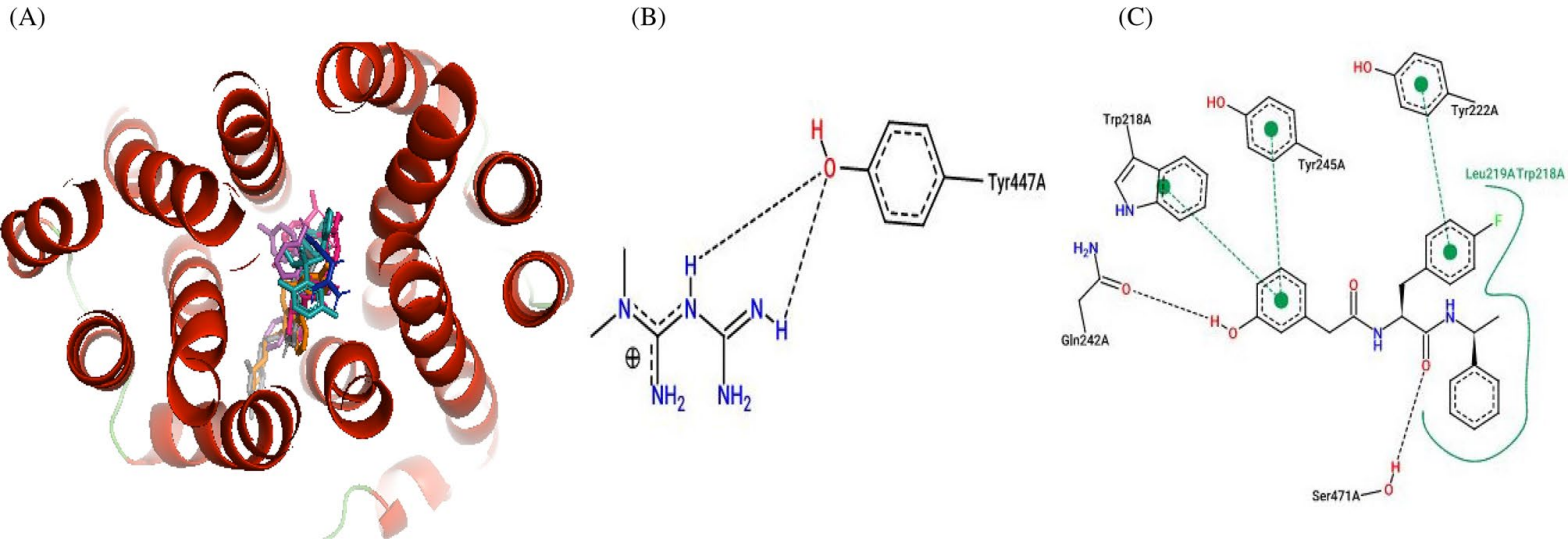
Ligand overlay for haplotype 3 *SLC22A2* protein. (**A**) Image was generated using Pymol with the first line anti-diabetic drug, Metformin, shown in blue. The four top binding ligands Dolutegravir, Glibenclamide, Vandetanib and Isavuconazole are shown in grey, hot-pink, orange and deep teal respectively. The 5RE inhibitor is indicated in magenta. (**B,C**) 2D PoseView illustration, showing interacting residues for Metformin and the 5RE inhibitor respectively—dotted black lines denote hydrogen bonds, solid green lines denote hydrophobic interactions and dotted green lines denote π stacking &/or aromatic interactions.

**Figure 9:**
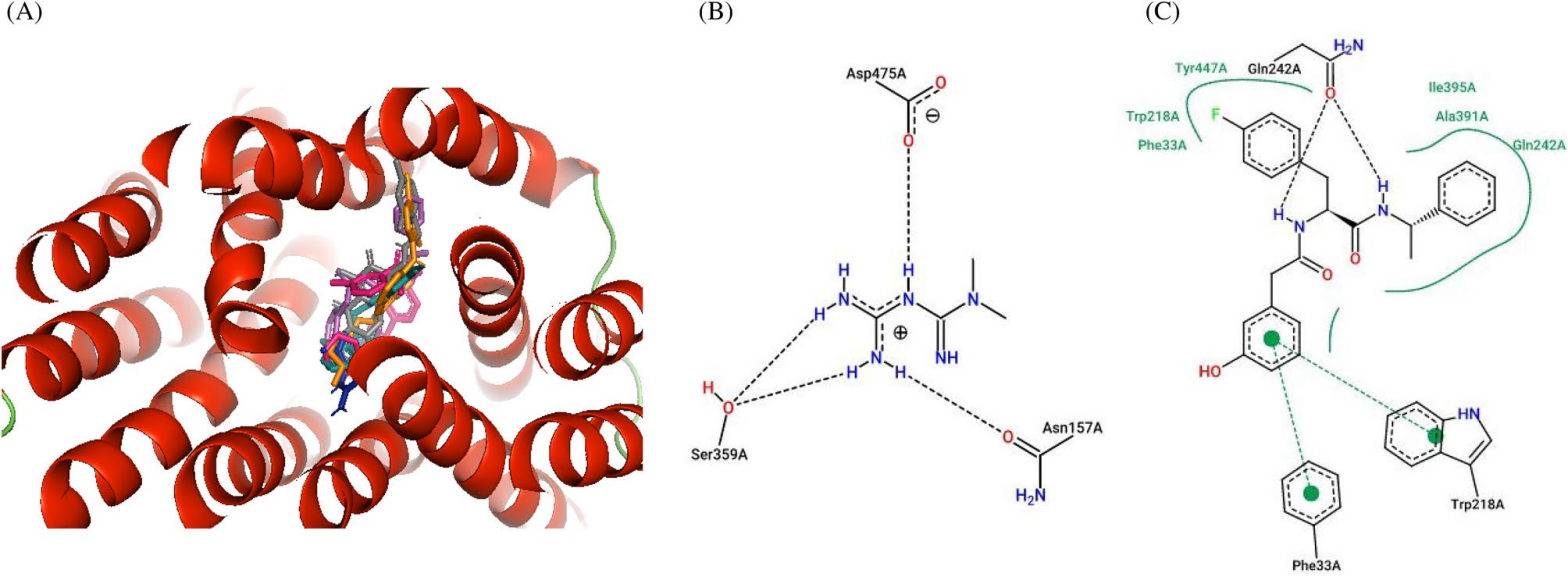
Ligand overlay for haplotype 4 *SLC22A2* protein. (**A**) Image was generated using Pymol with the first line anti-diabetic drug, Metformin, shown in blue. The four top binding ligands Isavuconazole, Dolutegravir, Glibenclamide, and Vandetanib are shown in deep teal, grey, hot-pink, and orange respectively. The 5RE inhibitor is indicated in magenta. (**B,C**) 2D PoseView illustration, showing interacting residues for Metformin and the 5RE inhibitor respectively—dotted black lines denote hydrogen bonds, solid green lines denote hydrophobic interactions and dotted green lines denote π stacking &/or aromatic interactions.

**Figure 10:**
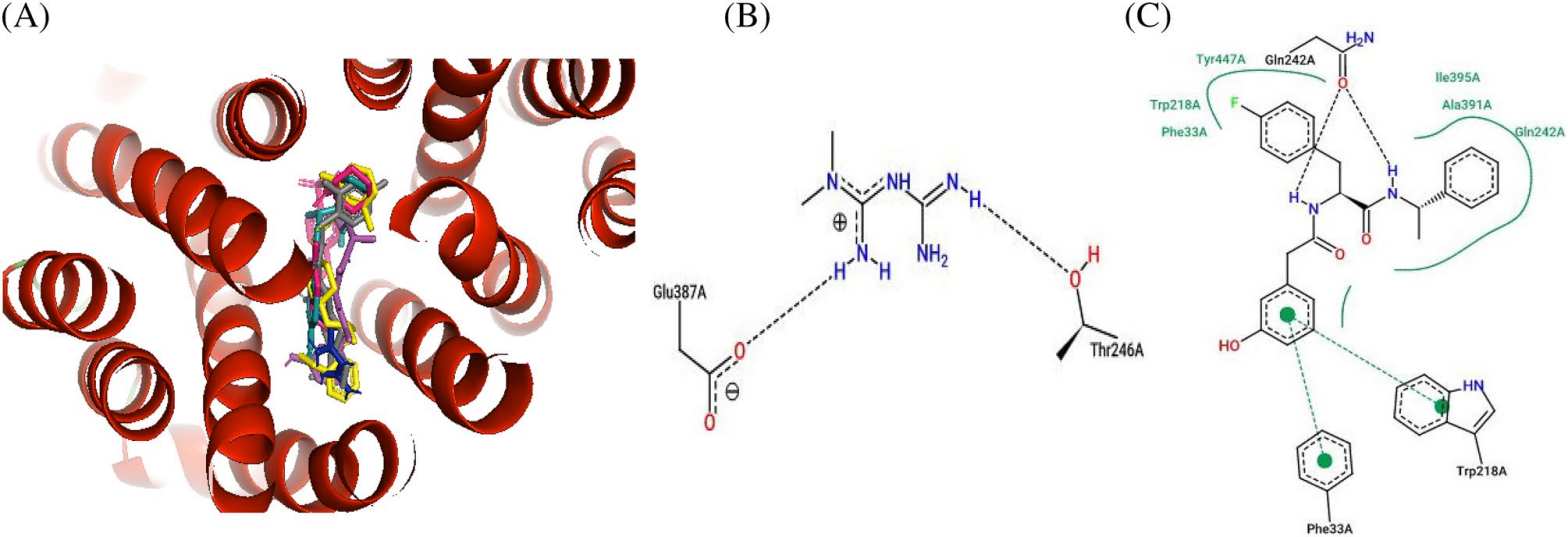
Ligand overlay for haplotype 5 *SLC22A2* protein. (**A**) Image was generated using Pymol with the first line anti-diabetic drug, Metformin, shown in blue. The four top binding ligands Dolutegravir, Glibenclamide, Isavuconazole and Ranolazine are shown in grey, hot-pink, deep teal, and yellow respectively. The 5RE inhibitor is indicated in magenta. (**B,C**) 2D PoseView illustration, showing interacting residues for Metformin and the 5RE inhibitor respectively—dotted black lines denote hydrogen bonds, solid green lines denote hydrophobic interactions and dotted green lines denote π stacking &/or aromatic interactions.

Important binding residues for the four top binding ligands and the 5RE inhibitor in haplotype 1 included Trp218, Tyr447 and Gln242 (Table 6 and Fig. 6). The residue Tyr447 was responsible for the strong binding of the four top binding ligands and the 5RE inhibitor in haplotypes 2 and 4 (Tables 7, 9 and Figs. 7, 9). In haplotype 3 aa residues Tyr245 and Ser471 featured as important for binding of the top four ligands and the 5RE inhibitor (Table 8 and Fig. 8). The residues Gln242 and Tyr447 were identified as essential in ligand binding in haplotype 5 (Table 10 and Fig. 10).

**Table 6:** Docking scores and number of interactions predicted for 11 drugs and 5RE inhibitor docked to the active site of the *SLC22A2* haplotype 1 (WT).

Ligand	Docking score (kcal/Mol)	Poseview calculated interactions		
		Hydrogen bonds	Hydrophobic interactions	π-Stacking &/or aromatic interactions
Isavuconazole	−9.8	2 (Thr444, **Ser471**)	2 (**Trp218, Tyr245**)	1 (**Tyr245**)
Dolutegravir	−9.7	1 (**Tyr37**)	1 (Lys215)	1 (**Tyr447**)
Vandetanib	−9.7	2 (**Tyr37**, Thr444)	2 (Tyr362, **Tyr447**)	2 (Tyr362, Thr444)
Glibenclamide	−9.0	1 (**Tyr37**)	5 (**Tyr37, Trp218**, Gln242, **Tyr245**, Cys451)	1 (**Tyr37**)
Gliclazide	−9.0	2 (**Tyr37**, Tyr362)	3 (Phe33, **Trp218**, **Tyr245**)	1 (**Tyr245**)
Ranolazine	−8.2	1 (Ser471)	6 (Phe33, Tyr37, Trp218, Gln242, Trp355, Tyr447)	1 (Tyr37)
Trimethoprime	−6.9	2 (Trp218, Tyr241)	1 (Tyr37)	1 (Tyr245)
Ranitidine	−6.3	3 (Gln242, Thr246, Glu387)	3 (Trp218, Tyr245, Tyr362)	1 (Tyr245)
Cimetidine	−6.1	1 (Thr444)	2 (Gln242, Tyr245)	1 (Tyr245)
Metformin	−4.9	2 (Thr246, Glu387)	0	0
Creatinine	−4.1	0	0	0
5RE	−10.3	3 (**Tyr37, Tyr447, Ser471**)	2 (**Trp218, Tyr245**)	1 (**Tyr245**)

Interactions calculated using Poseview. Number in front of brackets is the total number of interactions observed. Three letter aa code: *Thr *threonine, *Trp* tryptophan, *Tyr* tyrosine, *Lys* lysine, *Gln* glutamine, *Cys* cysteine, *Phe* phenylalanine, *Glu* glutamine, *Ser* serine. The residue highlighted in bold shows similar interacting residue shared between the top binders: i.e. 5RE, Isavuconazole, Dolutegravir, Vandetanib, Glibenclamide and Gliclazide.

The study showed that Glibenclamide, Gliclazide, Dolutegravir, Isavuconazole, Vandetanib as well as Ranolazine to have stronger affinity for the *SLC22A2* transporter than Metformin and the other six compounds (Tables 6, 7, 8, 9, 10). In addition to this, Metformin showed only hydrogen bond interactions in all haplotypes (Tables 6, 7, 8, 9, 10). In all five haplotypes, the top four ligands, Metformin and the 5RE inhibitor bound in the active site (Figs. 6, 7, 8, 9 and 10).

**Table 7:** Docking scores and number of interactions predicted for 11 drugs and 5RE inhibitor docked to the active site of *SLC22A2* haplotype 2.

Ligand	Docking score (kcal/Mol)	Poseview calculated interactions		
		Hydrogen bonds	Hydrophobic interactions	π-Stacking &/or aromatic interactions
Dolutegravir	−9.8	1 (Tyr245)	2 (Trp218, Lys215)	1 (**Tyr447**)
Glibenclamide	−9.8	2 (Lys215, **Tyr447**)	3 (Trp218, **Tyr447**, Cys451)	2 (Trp218, **Tyr447**)
Isavuconazole	−9.2	0	2 (Gln242, Thr444)	1 (**Tyr447**)
Gliclazide	−8.8	2 (Tyr245, **Tyr447**)	1 (Tyr37)	1 (Tyr37)
Vandetanib	−8.8	0	2 (Lys215, Tyr447)	1 (**Tyr447**)
Ranolazine	−8.0	1 (Tyr447)	3 (Trp218, Tyr245, Cys451)	1 (Tyr245)
Trimethoprime	−6.4	2 (Gln242, Glu387)	1 (Trp218)	2 (Trp218, Tyr245)
Ranitidine	−5.6	1 (Glu387)	2 (Gln242, Tyr447)	1 (Tyr447)
Cimetidine	−5.6	2 (Tyr245, Glu387)	0	0
Metformin	−4.5	2 (Glu387, Glu448)	0	0
Creatinine	−3.8	1 (Thr444)	0	0
5RE	−9.5	2 (Thr444, **Tyr447**)	3 (Lys215, **Tyr447**, Ser471)	1 (**Tyr447**)

Interactions calculated using Poseview. Number in front of brackets is the total number of interactions observed. Three letter aa code: *Tyr* tyrosine, *Trp* tryptophan, *Lys* lysine, *Cys* cysteine, *Gln* glutamine, *Glu* glutamic acid, *Thr* threonine, *Ser* serine. The residues highlighted in bold shows similar interacting residues shared between the top binders: i.e. 5RE, Dolutegravir, Glibenclamide, Isavuconazole, Gliclazide and Vandetanib.

**Table 8:** Docking scores and number of interactions predicted for 11 drugs and 5RE inhibitor docked to the active site of *SLC22A2* haplotype 3.

Ligand	Docking score (kcal/Mol)	Poseview calculated interactions		
		Hydrogen bonds	Hydrophobic interactions	π-Stacking &/or aromatic interactions
Dolutegravir	−9.9	2 (Lys215, **Ser471**)	0	1 (Ile223)
Glibenclamide	−9.7	1 (**Ser471**)	5 (Leu219, Gly238, Gln242, Tyr447, Ser471)	1 (**Tyr245**)
Vandetanib	−9.3	3 (Lys215, **Ser471**, Ser472)	3 (Leu219, Ile223, Ser471)	0
Isavuconazole	−9.0	1 (Gln242)	2 (Tyr37, Tyr447)	4 (Tyr37, Lys215, Tyr362, Tyr447)
Gliclazide	−8.6	0	2 (Trp218, Tyr222)	1 (Tyr245)
Ranolazine	−8.4	3 (Tyr222, Arg235, Glu448)	4 (Trp218, Tyr222, Gln242, Tyr447)	1 (Tyr222)
Trimethoprime	−7.0	3 (Gly238, Gln242, Glu448)	1 (Tyr447)	1 (Tyr447)
Ranitidine	−6.0	2 (Ser164, Ser472)	2 (Tyr37, Trp218)	1 (Trp218)
Cimetidine	−6.3	2 (Ser164, Ser472)	0	0
Metformin	−4.7	1 (Tyr447)	0	0
Creatinine	−4.2	2 (Ser164, Ser472)	0	0
5RE	−10.0	2 (Gln242, **Ser471**)	2 (Trp218, Leu219)	3 (Trp218, Tyr222, **Tyr245**)

Interactions calculated using Poseview. Number in front of brackets is the total number of interactions observed. Three letter aa code: *Lys* lysine, *Ile* isoleucine, *Ser* serine, *Leu* leucine, *Gly* glycine, *Gln* glutamine, *Tyr* tyrosine, *Arg* arginine, *Glu* glutamic acid. The residues highlighted in bold shows similar interacting residues shared between the top binders: i.e. 5RE, Dolutegravir, Glibenclamide, Vandetanib and Isavuconazole.

**Table 9:** Docking scores and number of interactions predicted for 11 drugs and 5RE inhibitor docked to the active site of *SLC22A2* haplotype 4.

Ligand	Docking score (kcal/Mol)	Interactions		
		Hydrogen bonds	Hydrophobic interactions	π-Stacking &/or aromatic interactions
Isavuconazole	−10.4	2 (Tyr37, Ser471)	3 (Lys215, Leu219, **Tyr447**)	2 (Tyr37, **Tyr447**)
Dolutegravir	−10.1	1 (**Tyr447**)	1 (Lys215)	1 (**Tyr447**)
Glibenclamide	−9.7	0	2 (Tyr37, **Tyr447**)	1 (Tyr245)
Vandetanib	−9.4	0	3 (Tyr37, Thr444, **Tyr447**)	1 (**Tyr447**)
Gliclazide	−8.9	0	1 (Tyr37)	1 (Tyr37)
Ranolazine	−8.9	1 (Thr444)	5 (Phe33, Gln242, Tyr245, Thr444, Tyr447)	1 (Phe33)
Trimethoprime	−7.0	(Thr246, Thr444)	1 (Tyr447)	1 (Tyr447)
Ranitidine	−6.4	2 (Tyr37, Ser359)	2 (Tyr37, Tyr245)	2 (Tyr245, Tyr362)
Cimetidine	−6.4	3 (Tyr37, Ser359, Tyr447)	1 (Tyr447)	0
Metformin	−5.4	3 (Asn157, Ser359, Asp475)	0	0
Creatinine	−4.4	3 (Tyr37, Ser359, Tyr447)	0	0
5RE	−10.0	1 (Gln242)	5 (Phe33, Trp218, Ala391, Ile395, **Tyr447**)	2 (Phe33, Trp218)

Interactions calculated using Poseview. Number in front of brackets is the total number of interactions observed. Three letter aa code: *Tyr* tyrosine, *Ser* serine, *Lys* lysine, *Leu* leucine, *Thr* threonine, *Phe* phenylalanine, *Asn* asparagine, *Asp* aspartic acid, *Ala* alanine. The residues highlighted in bold shows similar interacting residues shared between the top binders: i.e. 5RE, Isavuconazole, Dolutegravir, Glibenclamide, and Vandetanib.

**Table 10:** Docking scores and number of interactions predicted for 11 drugs and 5RE inhibitor docked to the active site of *SLC22A2* haplotype 5.

Ligand	Docking score (kcal/Mol)	Poseview calculated interactions		
		Hydrogen bonds	Hydrophobic interactions	π-Stacking &/or aromatic interactions
Dolutegravir	−10.0	2 (Thr246, Thr444)	1 (Tyr37)	1 (Tyr37)
Glibenclamide	−9.5	1 (Ser471)	3 (Tyr37, **Gln242**, **Tyr447**)	1 (**Tyr447**)
Isavuconazole	−9.3	1 (Thr444)	3 (Lys215, Trp219, **Tyr447**)	3 (Tyr37, Lys215, **Tyr447**)
Ranolazine	−9.2	4 (Tyr37, Thr246, Tyr362, Thr444)	4 (Phe33, Lys215, Tyr245, **Tyr447**)	1 (Tyr37)
Vandetanib	−8.9	0	3 (Gln242, Tyr362, Tyr447)`	2 (Tyr362, Tyr447)
Gliclazide	−8.6	2 (Tyr37, Tyr447)	2 (Tyr37, Tyr245)	1 (Tyr37)
Trimethoprime	−7.4	2 (Tyr447, Glu448)	1 (Tyr447)	1 (Tyr447)
Ranitidine	−6.3	(Thr246, Glu387, Thr444)	3 (Tyr37, Tyr362, Tyr447)	1 (Tyr362)
Cimetidine	−6.3	3 (Tyr37, Thr246, Tyr447)	1 (Tyr447)	0
Metformin	−5.3	2 (Thr246, Glu387)	0	0
Creatinine	−4.5	3 (Thr246, Tyr447, Glu448)	0	0
5RE	−9.7	1 (**Gln242**)	6 (Phe33, Trp218, **Gln242**, Ala391, Ile395, **Tyr447**)	2 (Phe33, Trp218)

Interactions calculated using Poseview. Number in front of brackets is the total number of interactions observed. Three letter aa code: *Thr* threonine, *Tyr* tyrosine, *Ser* serine, *Lys* lysine, *Phe* phenylalanine, *Gln* glutamine, *Glu* glutamic acid, *Ala* alanine, *Ile* isoleucine. The residues highlighted in bold shows similar interacting residues shared between the top binders: i.e. 5RE, Dolutegravir, Glibenclamide, Isavuconazole, and Ranolazine.

## Discussion

3D molecular modelling is routinely used in the discovery, development and design of drugs. Statistical data obtained from multiple protein conformations are considered more reliable than data obtained from single static structures, since proteins can occur in different conformations^50^. The predicted 3D protein model of *SLC22A2* was built using a template generated from multiple aa sequence alignments using AlphaFold 2 (Fig. S1). The constructed 3D protein model of *SLC22A2* successfully satisfied several quality checks suggesting the protein model is reliable and accurate for use in molecular dynamic simulations and docking studies (Fig. 1). The predicted docking scores of ligands ranged between −10.4 and −3.8 kcal/Mol across haplotypes (Tables 6, 7, 8, 9 and 10). Larger ligands (such as: Dolutegravir, Isavuconazole, Ranolazine, Trimethoprim, Vandetanib, Glibenclamide and Gliclazide) tend to show higher docking scores in comparison to smaller ligands (such as: Metformin, Cimetidine, Ranitidine and Creatinine). This can be attributed to larger ligands having more residues available to interact with in the binding pocket. Thus, it was observed that Metformin which is a small ligand, tends to have a lower docking score in comparison to its counter-parts Glibenclamide and Gliclazide in all haplotypes (Tables 6, 7, 8, 9 and 10). Glibenclamide and Gliclazide are second-generation sulfonylureas used in the treatment of T2DM. Mutations in the *CYP2C9* gene have been shown to influence the efficacy of their function^45,46^. They are commonly used in combination therapy with Metformin but can also be used as monotherapy. Combination therapy of Metformin and Glibenclamide as well as Metformin and Gliclazide has been shown to be more effective in the management of T2DM in patients who have not responded effectively to monotherapies available^51–53^.

The MD results showed that haplotypes 1, 2, 3 and 4 had higher RMSD and RMSF values suggesting they were less stable and more flexible compared to haplotype 5 being more stable based on lower RMSD and RMSF values. However, in contrast to the binding free energy haplotype 2 showed a higher average number of hydrogen bonds and non-bonded interaction energy with 5RE compared to haplotype 1, whilst haplotypes 3 and 5 only showed the least amount of average number of hydrogen bonds and non-bonded interaction energy compared to haplotype 1 and 4. The non-bonded interaction energy should be interpreted with caution as it excludes the polar solvation terms and solvent accessible surface area terms which is included in the free energy of binding calculation. Analysis of the POPC lipid bilayer indicated that the lipid structures for all five haplotype systems remained stable throughout the simulation, based on area per lipid and deuterium order parameters. Haplotypes 1, 2, 3 and 5 showed very little difference in membrane thickness with only haplotype 4 displaying smaller membrane thickness and larger lateral diffusion values suggesting higher mobility of lipids resulting in stronger POPC and protein interaction. Additionally, haplotypes 2, 3 and 4 showed higher binding affinity to the POPC lipid bilayer based on the number of hydrogen bonds and non-bonded interaction energy while haplotype 5 displayed weaker binding affinity to the POPC lipid bilayer.

Furthermore, haplotypes 4 and 5 also demonstrated the highest number of interactions with inhibitor 5RE and free binding energy compared to haplotype 1. For the docking of metformin to the five haplotypes again haplotype 4 and 5 higher docking scores compared to haplotype 1 and only haplotype 4 showed slightly higher number of interactions with haplotype 4 residues. Overall the docking results for the top four compounds to haplotypes 4 and 5 showed higher docking scores and in some instances higher number of interactions compared to haplotype 1. However, the binding interactions observed between proteins and ligands needs to be validated experimentally using X-ray or Cryo-EM methods. One of the most common SNPs of *SLC22A2* is rs316019 (A270S variant) which displays a minor allele frequency (MAF) of 80% in all ethnicities and has been associated with varied transport activity^54^. Sajib et al (2018) demonstrated through *in silico* analysis that the 270A variant of *SLC22A2* (i.e. WT) was better at binding Metformin and other drugs in comparison to the 270S variant (i.e. haplotype 2) since it had a more open and wider active site conformation^20^. Furthermore, three studies by Yoon et al. (2013), Wang et al. (2008) and Song et al. (2008) demonstrated individuals with the haplotype 2 genotype (270S) to produce a lowered Metformin clearance rate in comparison to individuals with the WT haplotype in Korean and Chinese subjects^55–57^. The above-mentioned studies could imply that the reduced Metformin clearance observed in the 270S genotype can be attributed to the smaller active site (as predicted by Sajib et al (2018)) making Metformin binding more efficient.

However, in contrast, Chen et al. demonstrated the 270S variant to produce a higher clearance of Metformin compared to the WT in Caucasian and African American ancestries^58^. In addition to this, Dujíc et al., Christensen et al. and Tzvetkov showed no statistically significant association between the 270S variant and glycaemic response to Metformin monotherapy^59–61^. The data generated herein demonstrates that haplotypes 4 and 5 could possibly have a stronger affinity for ligands as shown in our MD study with 5RE binding free energy, suggesting Metformin clearance can be reduced based on a higher number of interactions. Regardless of the contradiction in data, in population groups where polymorphism(s) may influence the response to Metformin therapy attention should be given to the phenotypic response of individual patients.

Haplotype 4 has the aa substitution (A270S and K432Q) and 5 (R400C) with substitutions A270S and R400C predicted to be deleterious and damaging to the protein function by SIFT and PolyPhen2 algorithms (Table 2). If we assume, that haplotypes 4 and 5 are most likely to cause a slower Metformin clearance rate because of their stronger affinity for ligand(s) in comparison to haplotype 1, individuals with these haplotypes are most likely to develop complications associated with Metformin toxicity. Drugs of the biguanide class, i.e. Metformin, if accumulated in the body cause an increase in plasma lactate level in a concentration-dependent manner by inhibiting mitochondrial respiration^20,62,63^. This could ultimately result in developing a life-threatening condition known as metformin-associated lactic acidosis (MALA)^62,64^. Therefore, dose adjustments of Metformin may prove beneficial for patients of haplotypes 4 and 5 in order to reduce the toxicity of Metformin and improve its efficacy. Trimethoprim has been shown to significantly reduce the clearance of both Metformin and Creatinine^65^. Creatinine is a by-product of muscle metabolism and creatinine serum levels are used as an indirect indicator of kidney function. Since Creatinine is a substrate of *SLC22A2* secreted into the urine, the binding of inhibitors to *SLC22A2* protein can cause a rise in serum creatinine levels that may be incorrectly interpreted as impaired kidney function^54^.

The findings in this study suggest haplotypes 4 and 5 have a higher affinity for drug binding based on binding free energy values and interaction analysis. However, a limitation in this study is the exclusion of entropy within binding free energy calculations using the g_mmpbsa tool and not including implicit membrane solute dielectrics for accurate polar solvation energy calculations. This will be considered in future studies. In some instances, such as with Metformin, the increased binding affinity can prove detrimental to the patient resulting in Metformin toxicity. Provided that organic cationic drugs are also used to treat various cancers and HIV, the results generated herein can be used to expand pharmacogenetic information available for African-specific populations, potentially impacting healthcare on the African continent. Future investigations will include cellular uptake analysis of Metformin to determine each haplotype’s transport activity identified for the *SLC22A2* gene in the Xhosa population of South Africa.

## Conclusions

In this study, the structural and functional effects of mutation(s) present within haplotypes were evaluated within the *SLC22A2* protein. The protein models generated for each haplotype of the *SLC22A2* gene indicated that haplotypes 4 and 5 of the Xhosa population of South Africa might impair the transport of Metformin via the *SLC22A2* protein transporter. In addition to this, we identified other drugs which may outcompete or inhibit Metformin transport via *SLC22A2*. We also observed, that larger ligands tend to preferentially bind in the conserved active site location of *SLC22A2*. Based on these evaluations, it can be deduced that T2DM patients should be cautious with the usage of over-the-counter drugs since it may lead to Metformin accumulation in the blood, resulting in increased lactate concentration and the increased likelihood of the development of MALA. It is recommended that future studies should experimentally validate the binding affinity of each haplotype for Metformin. In addition, results may be used to develop and/or improve pharmacogenomic profiling systems used for individualized therapeutic approaches employed in the treatment and management of T2DM.

## Supplementary Information

Supplementary Figures.

## Data Availability

The protein sequence analysed during the current study is available in the UniProt repository with accession number O15244. The genetic polymorphisms analysed during the current study are available in the dbSNP respository with accession number rs316019, rs8177516 and rs8177517.
