# Supplementary material for: The determination of the effect(s) of solute carrier family 22-member 2 (SLC22A2) haplotype variants on drug binding via molecular dynamic simulation systems
Source: Sci Rep. 2022 Oct 8;12:16936. doi: 10.1038/s41598-022-21291-4 (PMC9547889; doi:10.1038/s41598-022-21291-4)
Supplement: Supplementary file 1 — Supplementary Figures. [file 41598_2022_21291_MOESM1_ESM.docx]

**Supplementary information**

**
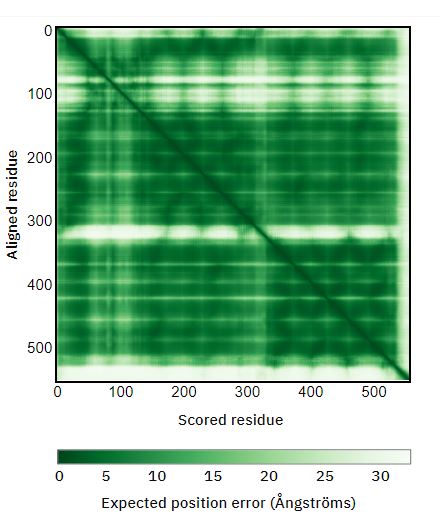
**


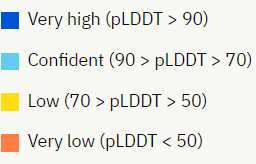

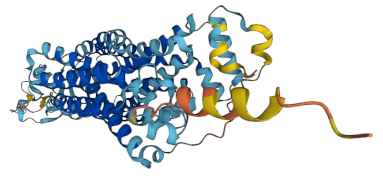


**Fig S1: AlphaFold 2 protein structure prediction for *SLC22A2*:** Predicted 3D structure of the *SLC22A2* protein based on the aa sequence with UniProtKB database accession number O15244. The per residue confidence pLDDT score (pLDDT – model’s predicted score on the IDDT-Cα metric) and the predicted aligned error (PAE) for the *SLC22A2* protein as determined by AlphaFold 2.


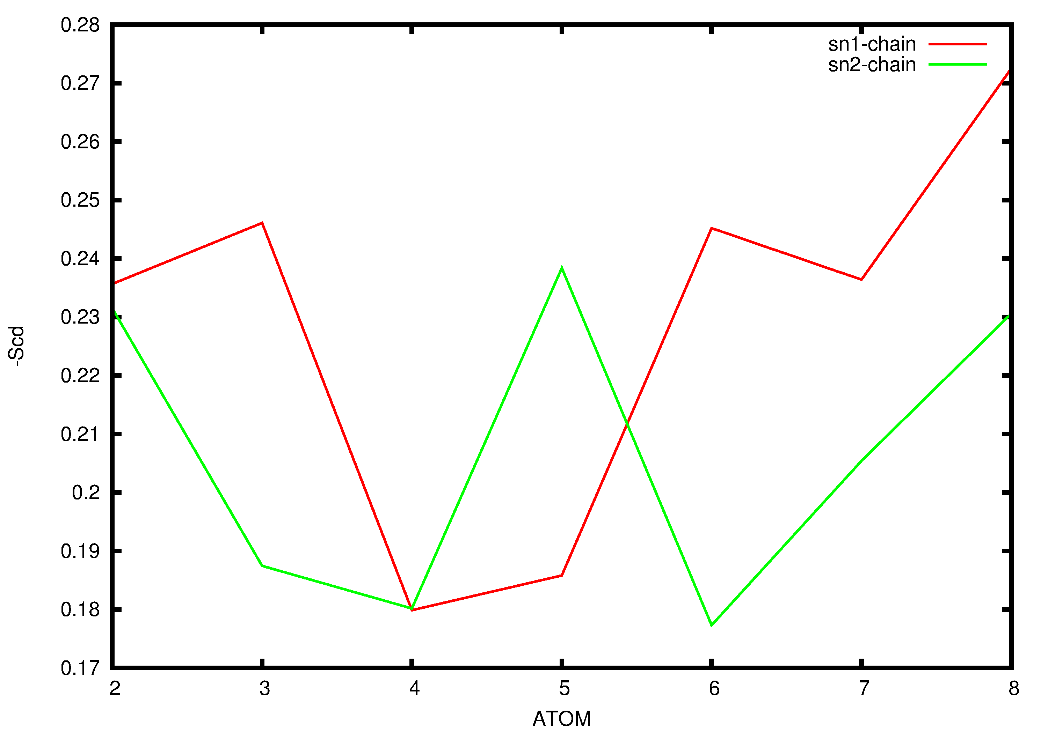


**Fig S2: Deuterium order parameters for the lipid acyl chains 1 and 2 for haplotype 1.**


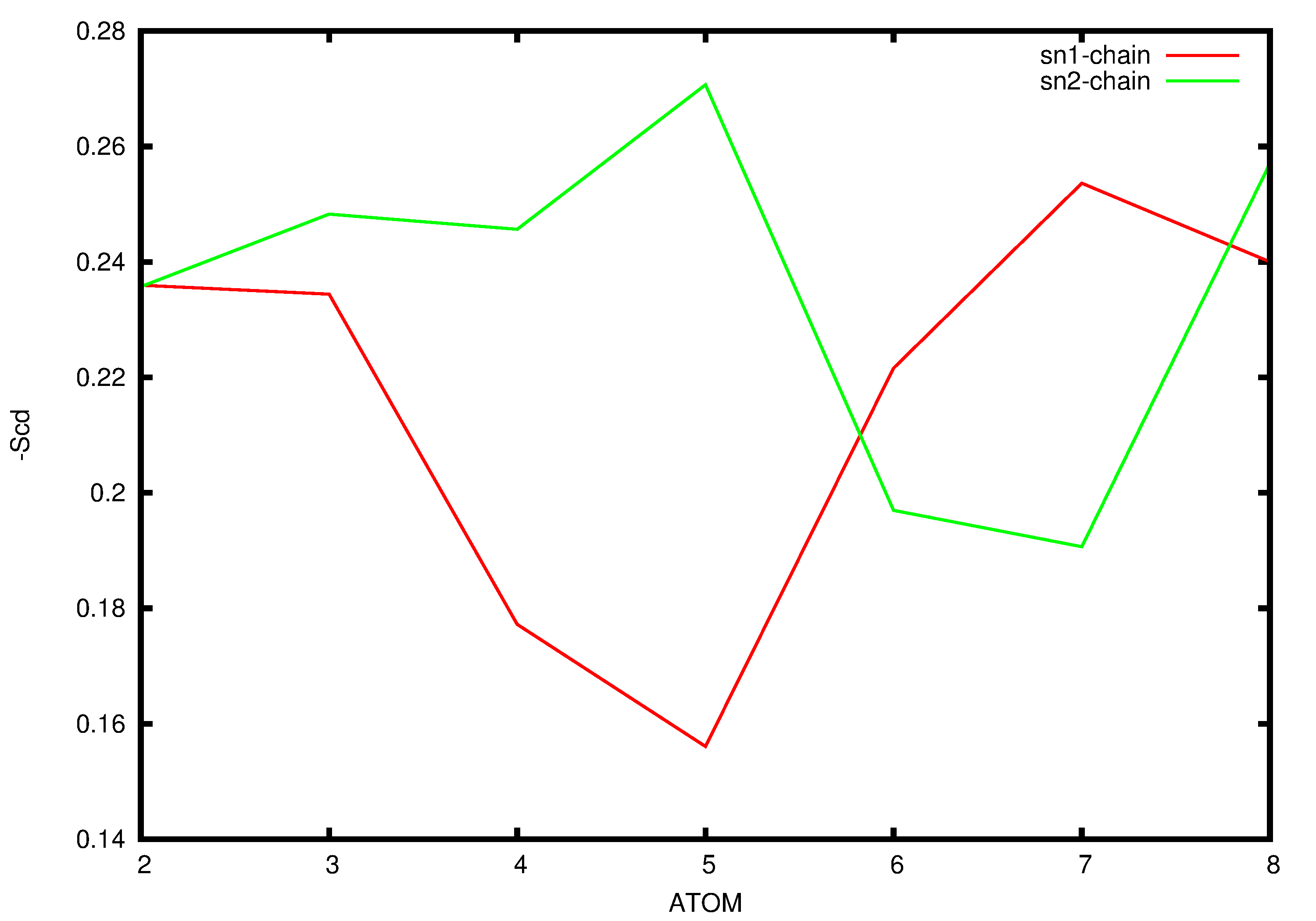


**Fig S3: Deuterium order parameters for the lipid acyl chains 1 and 2 for haplotype 2.**


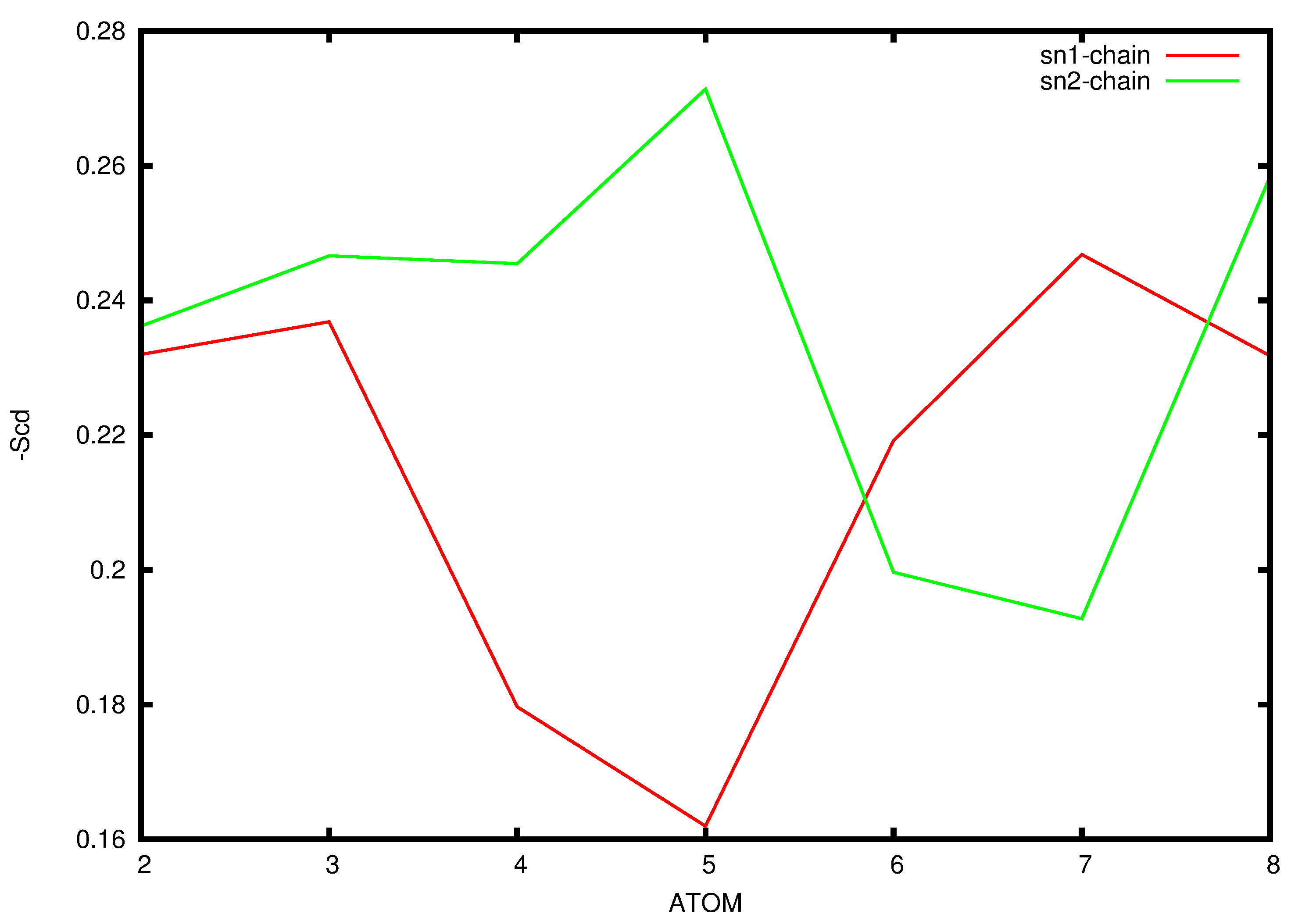


**Fig S4: Deuterium order parameters for the lipid acyl chains 1 and 2 for haplotype 3.**


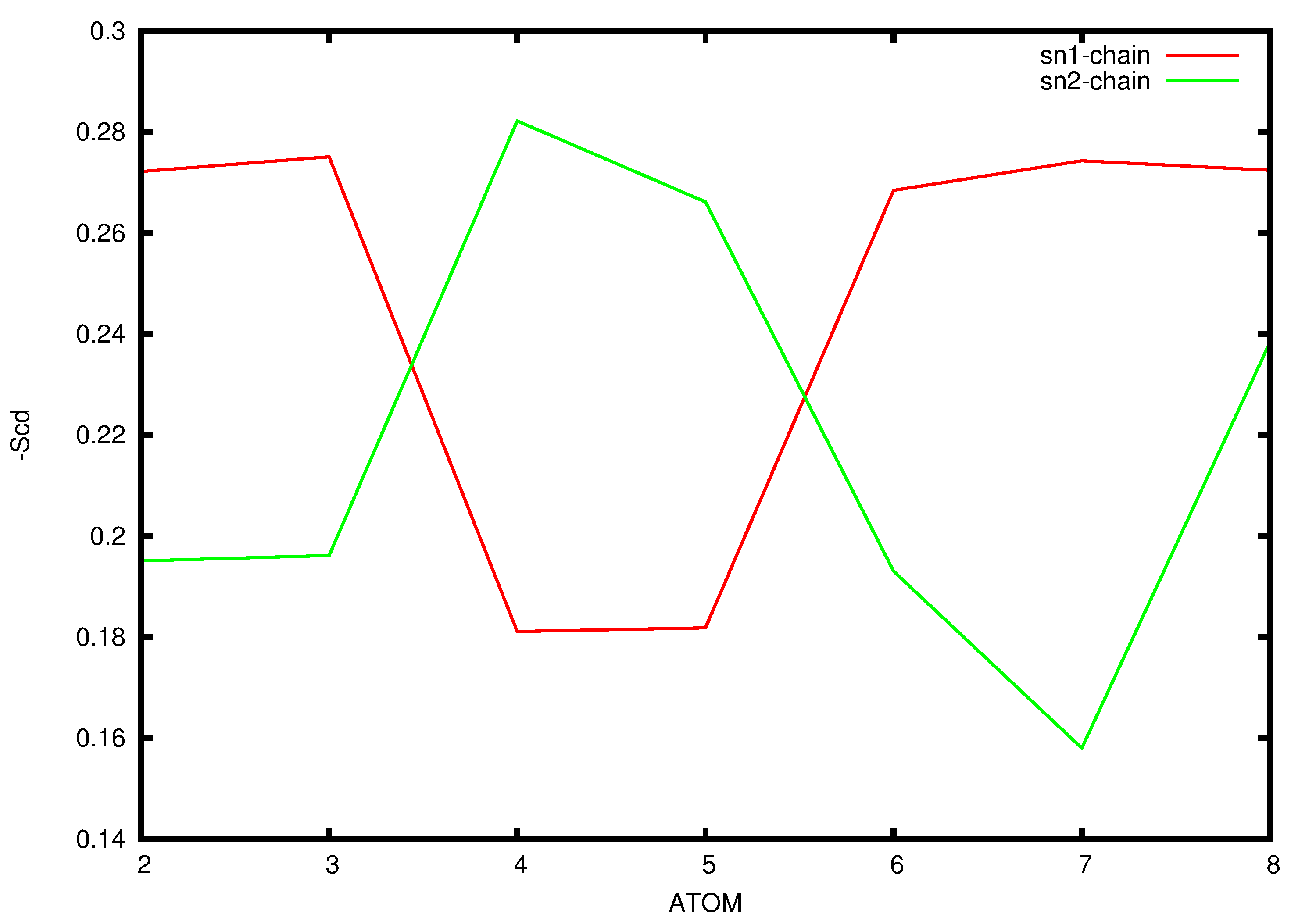


**Fig S5: Deuterium order parameters for the lipid acyl chains 1 and 2 for haplotype 4.**


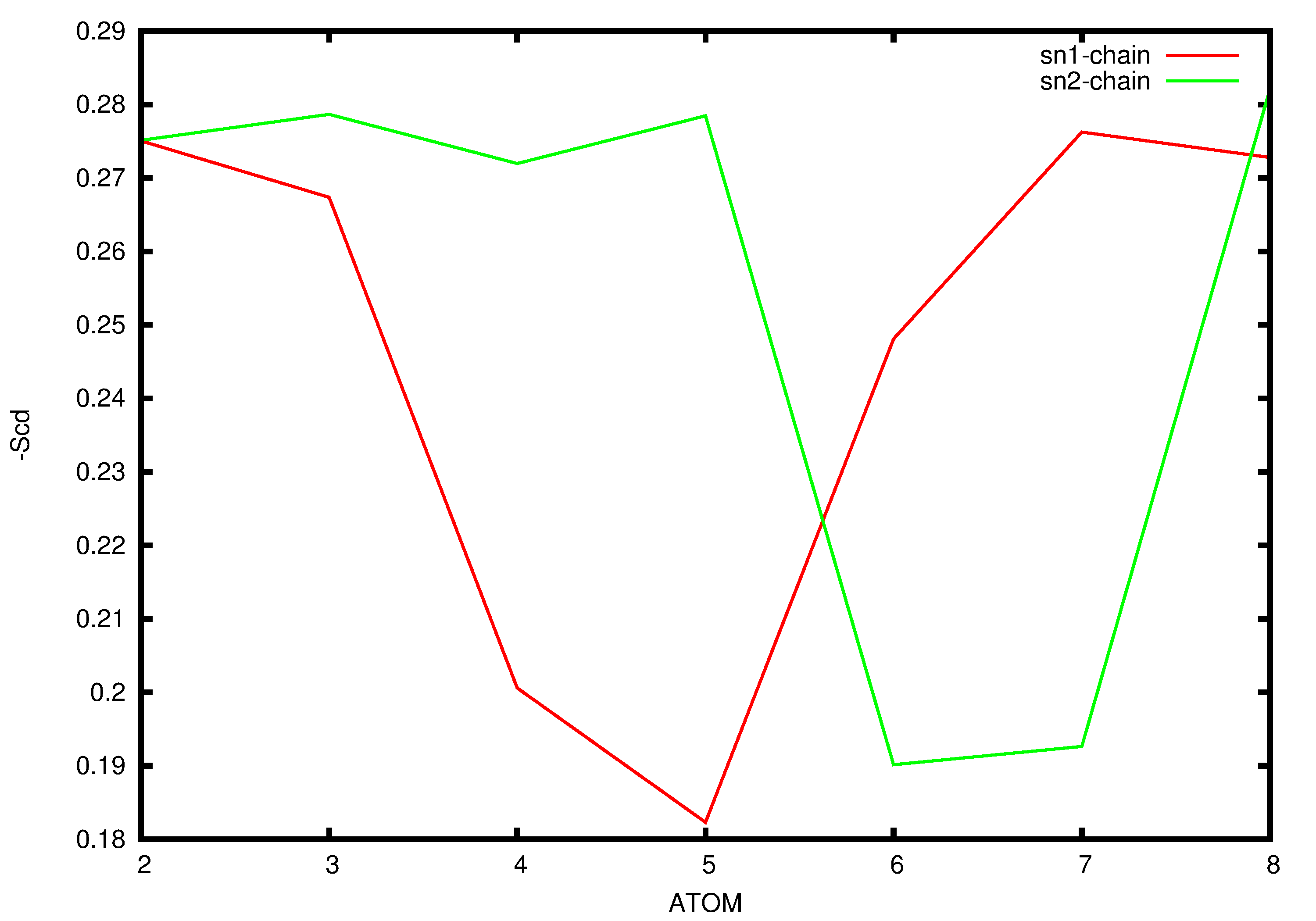


**Fig S6: Deuterium order parameters for the lipid acyl chains 1 and 2 for haplotype 5.**


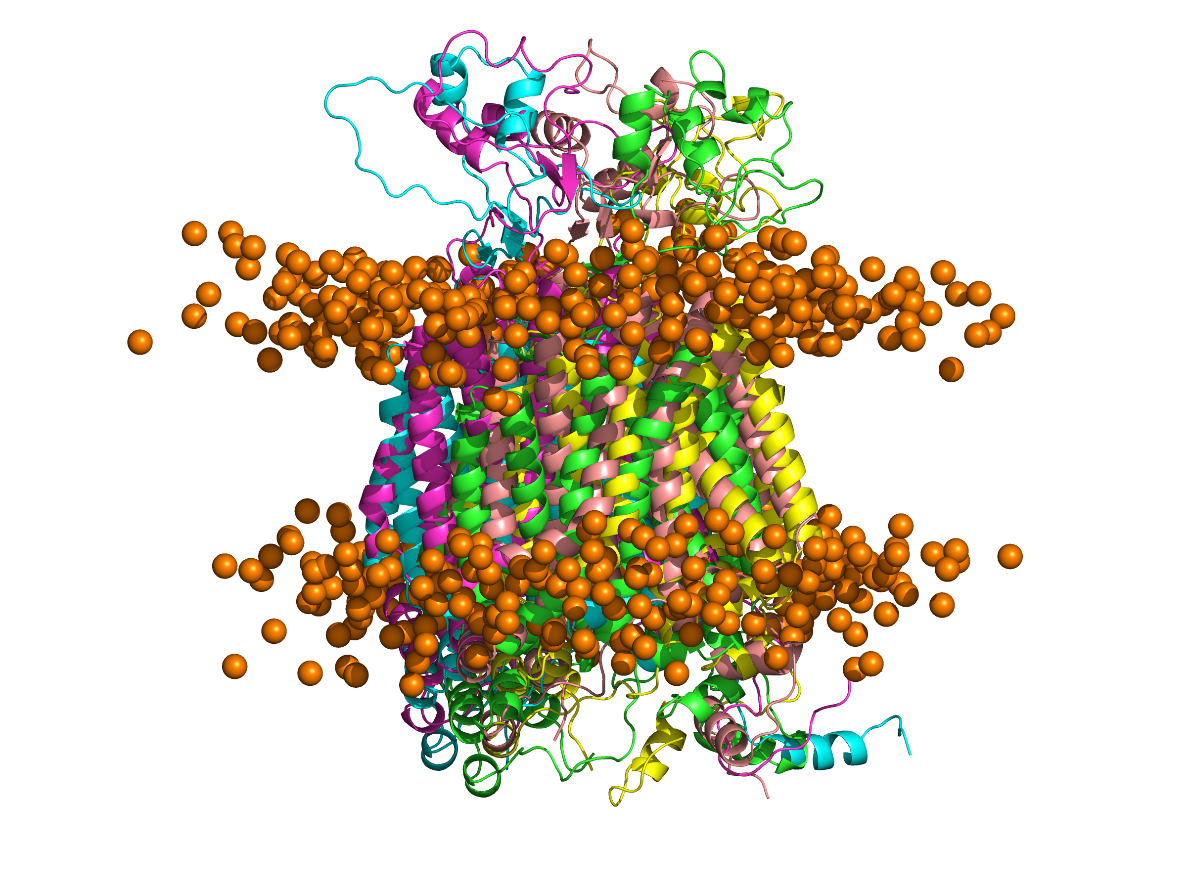


**Fig S7: Overlay of the final 200 ns snapshot structures for the five haplotypes.** Haplotype 1- green, haplotype 2- cyan, haplotype 3- magenta, haplotype 4- yellow, haplotype 5- light pink, phosphate lipid head groups shown as orange spheres.
